# Bactericidal Antibacterial Mechanism of Plant Synthesized Silver, Gold and Bimetallic Nanoparticles

**DOI:** 10.3390/pharmaceutics12111044

**Published:** 2020-10-30

**Authors:** Olufunto T. Fanoro, Oluwatobi S. Oluwafemi

**Affiliations:** 1 Centre for Nanomaterials Sciences Research, University of Johannesburg, Johannesburg 2028, South Africa; jolufunto@gmail.com; 2 Department of Chemical Sciences (Formerly Applied Chemistry), University of Johannesburg, P.O. Box 17011, Doornfontein, Johannesburg 2028, South Africa

**Keywords:** antibiotic resistance, bactericidal, metal nanoparticles, *Escherichia coli*, *Staphylococcus aureus*, cell membrane, DNA damage

## Abstract

As the field of nanomedicine develops and tackles the recent surge in antibiotic resistance, there is a need to have an in-depth understanding and a synergistic view of research on the effectiveness of a metal nanoparticle (NP) as an antibacterial agent especially their mechanisms of action. The constant development of bacterial resistance has led scientists to develop novel antibiotic agents. Silver, gold and its bimetallic combination are one of the most promising metal NPs because they show strong antibacterial activity. In this review we discuss the mode of synthesis and the proposed mechanism of biocidal antibacterial activity of metal NPs. These mechanisms include DNA degradation, protein oxidation, generation of reactive oxygen species, lipid peroxidation, ATP depletion, damage of biomolecules and membrane interaction.

## 1. Introduction

The continuous emergence of resistant strains of bacteria to current antibiotics is a serious challenge in public health and threat to human existence. Thus, there has been strong impetus to develop new bactericides. This makes current research in bactericidal nanomaterials of high interest [1]. Antimicrobial resistance (AR) has been recognized as one of the major threats to global health and economy. Its effect is non-discriminatory to geographical locations, race, age or social status, thus putting at risk the gains of the Millennium Development Goals and threatens the achievement of the Sustainable Development Goals. Based on this, the World Health Organization (WHO) has considered some bacteria as high risk pathogens with a high focus on the ESKAPEE pathogens (*Enterococcus faecium*, *Staphylococcus aureus*, *Klebsiella pneumoniae*, *Acinetobacter baumannii*, *Pseudomonas aeruginosa*, *Enterobacter* spp. and *Escherichia coli*), which play a prominent role in this global epidemic [2,3,4]. The inherent problem in AR is that it is a natural phenomenon but several factors such as abuse and irrational use of antibiotics by humans have substantially expedited the process, causing multidrug-resistant (MDR) infection, which substantially lessens the therapeutic efficiency of antibiotics. This has given rise to severe consequences such as increased medical expenses, an overburdened public health system, prolonged hospitalization and increased mortality rates [4,5]. The rate at which bacteria develops antibiotic resistance surpasses the discovery and development of combative new antibiotics. The lead reasons are due to the bureaucracy of regulations, scientific challenges, regulatory issues and inadequate profitability from the new products [6]. Evidently, it is important to explore innovative strategies to fight antimicrobial resistance infection. Owing to the excellent antibacterial performance and high specific surface area, NPs have been commonly studied by researchers globally as a hopeful antimicrobial agent. In addition, bacteria rarely develop resistance to them due to the multifaceted antibacterial mechanism of NPs [7]. Metal nanoparticles (MNPs) are significant nanomaterials with excellent physiochemical, photothermal, magnetic and electrical properties [8,9,10,11]. They have been used as potent antimicrobial agents for bacterial infection detection, diagnosis and treatment [10,11,12,13]. The antibacterial effect of MNPs is based strongly on their size, shape surface chemistry and inherent constituents of their structure [14,15,16]. To date, various methods have been used to synthesize MNPs of different shapes such as rods, stars, spheres, cubes, etc. [17,18,19]. Amongst metallic elements, gold and silver as “noble” metals have been the focus of various research areas in recent years especially in biomedical applications for antibacterial activity. This is because of their potential to decrease or eradicate the development of more resistant bacteria because they aim at multiple biomolecules at once, preventing the development of resistant strains [20]. Several authors have discussed the green synthesis of NPs and their biological applications. However, in this review, the focus is on the synthesis strategies and possible antibacterial mechanism of specific MNPs such as silver (Ag), gold (Au) and their bimetallic synthesized from plants. This is to give a synergistic view of the efficacy of plant mediated MNPs for the prospect of antibiotic development.

## 2. Biogenic Synthesis

The common methodology used in the synthesis of metal colloidal dispersion is via reduction of metal complexes. Prior to the need for green protocols, chemical agents such hydrazine hydrate and sodium borohydride have been used as reducing and capping agents, but their toxic effects have made them undesirable for biological applications. The biological approach to the synthesis of MNPs has resulted in non-toxic, simple and stable green routes that leads to translational research.

**Figure 1:**
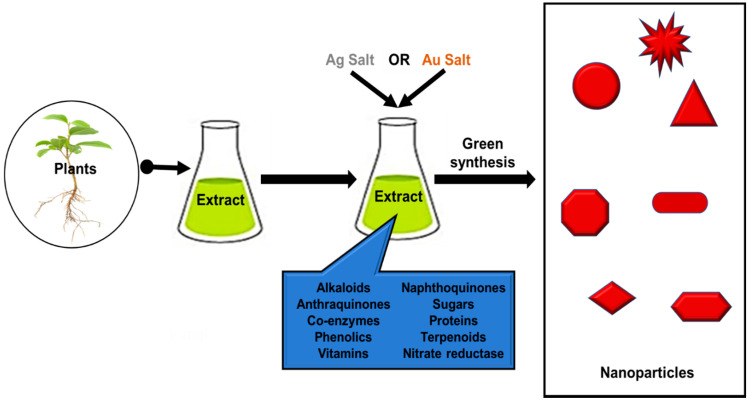
Schematic representation for the green synthesis of metal nanoparticles using different biomolecules.

Biological conventions for synthesis of silver nanoparticles (AgNPs) and gold nanoparticles (AuNPs) are through the use of nature’s biolaboratory such as plant, microorganisms, alga, carbohydrates and biopolymers [21,22]. They contain naturally occurring biomolecules that play significant roles in the reduction and capping of NPs [23,24]. Thus, they are deemed a green, sustainable and efficient route for the biosynthesis of NPs owing to their benign and environmentally friendly nature. For biogenic synthesis, simply biological extracts are mixed with the metal salt solutions and the effect of different parameters such as the concentration of the metal salt and extract, pH, temperature, time and radiation are studied [25,26]. Biological extracts comprise of biomolecules like terpenoids, enzymes, coenzymes, phenolics, alkaloids, amino acids, sugars, proteins, etc., that oxidize metal salts from positive oxidation state to the zero oxidation state (Figure 1). These biomolecules in turn determine the size and size distribution of MNPs. A strong reductant in biological extracts cause a rapid reaction rate and facilitates the formation of smaller NPs. Thus, a narrow size distribution is seen if there is a rapid reduction of metal salt by biomolecules, due to the formation of a new nuclei or secondary nuclei. On the other hand, if it happens that the secondary nucleation is suppressed over the primary one, a slow reaction may occur [27]. In addition, these biomolecules serve as a secondary option to form a monolayer on the surface of NPs to prevent agglomeration [18].

Recent studies of biological extract mediated synthesis of NPs confirm the surface functionalization of nanoparticles with biomolecules, which also improve their bactericidal activity. Thus, improved antimicrobial activity of green synthesized MNPs are due to the biomolecules attached on the surface of NPs [28,29,30]. Table 1 shows an analysis of the discussed plant synthesized NPs.

### 2.1. Silver Nanoparticles

Silver is known for its inhibitory effect on various pathogenic bacteria found in medical and industrial processes. Biosynthesis of silver nanoparticles has been shown as an easier and benign method compared with chemical methods [31]. A unique property of AgNPs is their large surface area and the fractions found on their surface atoms, which is significant in therapeutic applications [32,33].

Different parts of plant extracts have been used in the synthesis of AgNPs using silver ions as substrates. Dhand et al. [34] reported the synthesis of highly stable crystalline spherical AgNPs (20–30 nm) by exposing the hydroalcoholic extract of *Coffea arabica* to silver nitrate solution. They reported smaller particle size with a higher concentration of silver nitrate at 0.1 M within 2 h of synthesis at room temperature. The as-synthesized AgNPs had an inhibitory effect against *E*. *coli* and *S. aureus*. Khali et al. [35] reported the synthesis of spherical AgNPs using the aqueous extract of the olive leaf. They observed that smaller size NPs were recorded at an alkaline pH of 8 rather than an acidic pH of 3. Aqueous extract of *Ocimum sanctum* and its bioactive compound, quercetin, were used for the synthesis of spherical AgNPs by Jain and Mehata [36]. It was reported that quercetin produced a smaller particle size of 11.35 nm and a narrow plasmon peak as compared to the whole leaf extract of *Ocimum sanctum* with a particle size of 14.6 nm. This suggests that quercetin is a strong redundant present in *Ocimum sanctum*. Leela et al. [37] studied different leaf extracts of plants, namely, *Helianthus annus*, *Basella alba*, *Oryza sativa*, *Saccharu icinarum*, *Sorghum bicolar*, and *Zea mays* They found that among all the tested plant extracts, *H. annus* had the strongest potential for rapid reduction of silver ions. Shaik et al. [38] demonstrated the effect of a different volume of the *Origanum vulgare* extract in the synthesis of a different size of AgNPs. Increasing the volume of the extract produced smaller NPs, which had a bactericidal effect on both Gram positive (+ve) and Gram negative (−ve) bacteria. Padalia et al. [39] used the aqueous extract of the *Tagetes erecta* flower to synthesis predominantly spherical and hexagonal AgNPs. The as-synthesized AgNPs coupled with commercial antibiotics had a better antibacterial effect than using the commercial antibiotics alone. In our group, we reported the synthesis of spherical AgNPs using the aqueous extract of *Combretum erythrophyllum* leaf and it was reported to have strong antibacterial activities against *Staphylococcus* species implicated in dermatological infections [18]. Nouri et al. [40] used the aqueous extract of the *Mentha aquatica* leaf extract to synthesize spherical AgNPs. The results showed the significant effect of the ultrasound during the synthesis to produce smaller AgNPs (8 nm) with enhanced antibacterial activity by lowering the minimum inhibitory concentration (MIC) as compared to those synthesized with the hydrothermal method. In recent times, bioinspired synthesis of polygonal AgNPs using the ethyl acetate fraction of the alcoholic extract of pomegranate leaves has been reported. The ethyl acetate fraction had a smaller particle size compared to that of the aqueous extract [41]. This suggests that the ethyl acetate fraction had a strong redundant that produced smaller NPs.

### 2.2. Gold Nanoparticles

AuNPs are desired noble MNPs because of their possible usage in diverse fields of science and engineering such as gene expression and therapy, catalysis, optics, nanoelectronics, nanomedicine and disease diagnosis [42]. Increased toxicity concerns over chemical synthesis routes have drawn considerable interest toward green synthesis of AuNPs. Muthukumar et al. [43] biosynthesized AuNPs using the leaf extracts of *Carica papaya* and *Catharanthus roseus*. The mixture of both extracts produced predominantly spherical AuNPs and they had increased antibacterial effects compared with the AuNPs synthesized from the individual plant extract. Patra et al. [44] reported the formation of spherical AuNPs with strong antibacterial effects when synergized with rifampicin and kanamycin. Similarly, other researchers confirmed the synthesis of gold nanoparticles using different plant parts such as the stem extract of *Cannabis sativa* (Indian hemp) [45], fruit extracts of *Amomum villosum* [46] and leaf and fruit extract of *Pistacia atlantica* [47]. Hamelian et al. [48] reported the formation of AuNPs from the aqueous extract of thyme. Awad et al. [49] reported the use of *Olea europaea* fruit extract *and Acacia nilotica* husk extract mixture as a bioreductant for the synthesis of AuNPs, which showed a significant antibacterial effect against *K. pneumoniae*, and *Pseudomonas* spp. As reported by Kumar et al. [50], *Croton caudatus Geisel* leaf extract was able to reduce chloroauric acid in twenty-five minutes to form stable and spherical AuNPs. The formation of gold nanowires was reported from the pulp extract of *Beta vulgaris*. The mechanism for the formation of the nanowires was by Brownian motion; small NPs dissolved in the solution, grew to larger ones and joined together via Brownian motion to form wire-like structures [51]. Very recently, Akintelu et al. [52] reported the synthesis of spherical and stable gold nanoparticles using pulp of *Garcina kola* at pH 7. Similarly, Wongyai et al. [53] studied the biosynthesis of AuNPs using the aqueous extract of *Cryptolepis buchanani* Roem to produce highly stable, small-sized AuNPs with a uniform spherical shape at pH 7. The stability confirmed by the zeta potential analysis was −30.28 mV.

### 2.3. Ag-Au Bimetallic Nanoparticles

**Figure 2:**
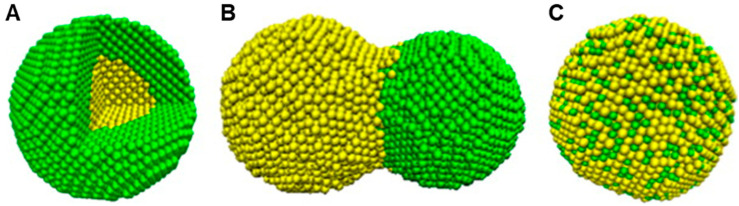
Schematic illustration of three mixing patterns in zero-dimensional bimetallic nanomaterials: (**A**) core–shell, (**B**) dumbbell and (**C**) alloyed (adapted with permission from [56], Elsevier, 2013).

**Figure 3:**
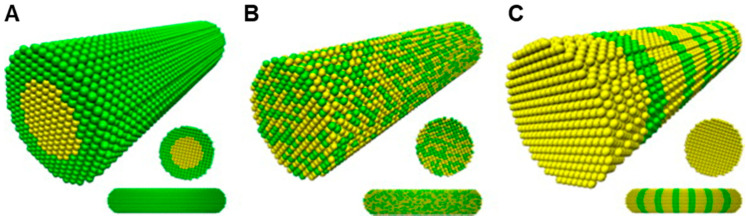
Schematic illustration of three mixing patterns in one-dimensional bimetallic NMs: (**A**) core–shell (**B**) alloyed and (**C**) bamboo-like (adapted with permission from [56], Elsevier, 2013).

Bimetallic nanoparticles (BMNPs) technically excel their mono metallic counterparts due to their improved electronic, optical and catalytic properties [54]. Aside from the morphological manipulations, the variations in the molar ratio of different components offers a diverse dimension in adapting the properties of BMNPs [54,55]. BMNPs, comes in various forms such as an alloy, core–shell and contact aggregate (dumbbell or bamboo-like). This is seen in the surface plasmon resonance peak either as a single (alloy) or multiple (core–shell/contact aggregate) [56,57] (Figure 2 and Figure 3). Among a wide range of BMNPs, silver and gold nanocompositions have gained significant advancement in drug delivery and nanomedicine [58]. Elemike et al. [57] reported synthesis of the Ag-Au alloy using the aqueous extract of *Solidago canadensis*. They observed the shape of the alloy to be similar to that of AuNPs alone. This was also confirmed by the higher concentration of Au in the EDX spectra. In a similar manner they also reported the synthesis of a nanoalloy Ag-Au BMNPs using the leaf extract of *Stigmaphyllon ovatum* [59]. Gopinath et al. [60] employed the use of *Gloriosa superba* for the synthesis of the Ag-Au nanoalloy. They observed that the BMNPs had a more efficient antibacterial activity effect on *Bacillus subtilis* than individual NPs. This was proposed to be due to the synergistic effect of the metals. Kumari et al. [61] reported the formation of nanoalloy Ag-Au BMNPs by using the juice of pomegranate seeds as a bioreductant. Recent studies by Emma [62] reported the production of nanoalloy Ag-Au BMNPs using a green approach by Arabic gum. It was observed that increasing the reaction temperature from 25 to 70 °C resulted in a well dispersed and smaller bimetallic NPs, which led to a size reduction from 6.5 to 3.1 nm. In another recent development, Gupta et al. [63] used aqueous leaf extract of *Moringa oleifera* for the biofabrication of the stable Ag-Au nanoalloy with a zeta potential of −36.7 mV.

**Table 1:** Biogenic synthesis of metal nanoparticles (MNPs) and bimetallic nanoparticles (BMNPs).

S/N	Sample	Biogenic source/Extraction Method	Bioactive Compound	NPs	Size (nm)	Shape	Ref.
1	*Coffea arabica*	Seed/ Ethanolic Extraction at 60 °C for 1 h	Phenolics	Ag	20–30 nm	Spheres and ellipsoidal	[34]
2	Olive tree	Leaf/Aqueous extraction by boiling for 10 min	Oleuropein	Ag	20–25 nm	Spheres	[35]
3	*Ocimum sanctum* and quercetin	Leaf/Aqueous extraction at 60 °C for 10 min	Quercetin	Ag	14.6 nm and 11.35 nm	Spheres	[36]
4	*Origanum vulgare*	Leaf/Aqueous extraction by reflux by for 4 h	Alkaloids, flavonoids, terpenoids	Ag	2–25	Spherical	[38]
5	*Tagetes erecta* (Marigold)	Flower/Aqueous extraction for 10 min	Flavonoids, saponins	Ag	46.11 nm	Spheres	[39]
6	*Combretum* *erythropyllum*	Leaf/Aqueous extraction at 90 °C for 1 h	Flavonoids	Ag	5–26 nm	Spherical	[18]
7	*Mentha aquatica*	Leaf/Aqueous extraction by sonication	Polyphenols, flavonoids		8 nm	Spheres	[40]
8	*Punica granatum*	Leaf/Ethanolic extraction for 48 h at room temperature	Polyphenols, flavonoids	Ag	20–40 nm	Polygonal	[41]
9	*Carica papaya* and *Catharanthus roseus*	Leaf/Aqueous extraction at room temperature	Papain, α-tocopherol, alkaloids, flavonoids	Au	6–18 nm	Spherical, Triangle, hexagonal	[43]
10.	*Citrullus lanatus* rind (Watermelon)	Fruit/Aqueous extraction for 10 min by boiling	Citrulline, proteins, carotenoids	Au	20–140 nm	Spheres	[44]
11	*Cannabis sativa* (Indian Hemp) Cortex and Xylem	Stem /Aqueous extraction for 10 min by boiling	Cannabinoids, terpenes, phenolics	Au	12–18 nm and 20–40 nm	Spheres, rod, Triangle, hexagonal	[45]
12.	*Amomum villosum* (Cardamom)	Fruit/Aqueous at 100 °C for 1 h via autoclave	***	Au	5–10 nm	Spheres	[46]
13	*Pistacia atlantica*	(Leaf and fruit)/Aqueous by boiling for 30 min	***	Au	50–60	Spheres	[47]
14	Thyme	Leaf/Aqueous by boiling for 30 min	***	Au	6–26 nm		[48]
15	*Olea europaea* fruit extract *and Acacia nilotica* husk	Fruit and husk/Aqueous extraction at room temperature	***	Au	44.96 nm	Spheres	[49]
16	*Croton caudatus Geisel*	Leaf/Aqueous extraction at 50 °C for 10 min	***	Au	20–50 nm	sphere	[50]
17	*Beta vulgaris* (Sugar beet)	Pulp/Aqueous purification	***	Au	50 nm	Nanowires	[51]
18	*Garcina kola*	Pulp/Aqueous extraction by boiling for 40 min	***	Au	18–38 nm	Spheres	[52]
19	*Cryptolepis buchanani*	Tea/Aqueous extraction at 60 °C for 15 min	Flavonoids, alkaloids, saponins, tannins	Au	11.1 nm	Spheres	[53]
20	*Solidago canadensis*	Leaf/Aqueous extraction at 80 °C	Flavonoids, quercetin, saponins	Ag-Au	15 nm	Spheres	[57]
21	*Stigmaphyllon ovatum*	Leaf	***	Ag-Au	14.9 nm	Spheres	[59]
22	*Gloriosa superba*	leaf/Aqueous extraction at 60 °C for 50 min	Superbine, colchicine, phytosterils, stigmasterin	Ag-Au	10–20 nm	Spheres	[60]
23	Pomegranate	Seed/Aqueous extraction	Phenolics	Ag-Au	12 nm	Spheres Rods Pentagonal	[61]
24	Arabic gum	Stems and branches of Arabic Senegal tree/Aqueous dissolution	Arabinose, rhamnose, glucoronic acid, arabinogalact-an–protein complex	Ag-Au	3.1 nm	Spheres	[62]
25	*Moringa oleifera*	Leaves/Aqueous extraction at 80 °C for 15 min	Niazimicin, 4-(α-L-rhamnosyloxy) benzyl isothiocyanate, β-sitosterol-3-O-β-D-glucopyranoside	Ag-Au	11–25 nm	Spheres Triangles Hexagonal	[63]

*** Not available.

## 3. Bacterial Resistance and Mutations

Antimicrobial resistance is a natural, intrinsic phenomenon, which can also be acquired or transferred in an effort to escape the actions of antimicrobial agents. Bacterial species have capabilities to resist or reduce the effect of antibiotic due to their natural inherent functional or structural features. [64,65]. The evolution of drug resistance occurs in a minimum of three phases namely, acquisition, expression and selection for microbes expressing those resistance genes. Foremost, bacteria gain resistance to one or more drugs by transduction, transformation and conjugation, which happens via horizontal gene transfer (HGT). Such antimicrobial agents threatened by HGT are β-lactams, fluoroquinolones, etc. [65,66,67,68]. Another way in which bacteria acquire a resistance gene is through spontaneous mutation of existing genes [69,70]. Multiple drug resistance (MDR) happens when bacteria with an existing drug resistance gene acquires resistance to another drug [65]. Secondly, in defense against exposure to antimicrobials, bacteria express the resistance gene [67]. Thirdly, resistance becomes prevalent when there is a suitable environment of growth for microorganisms that express resistance genes against the antibiotic. This conditional/selective pressure happens when the microorganisms are exposed to the antibiotic without elimination either by bactericidal or bacteriostatic effects of the antibiotic itself [64,66]. Use of a time-dependent antibiotic with long half-life and poor patient compliance can create the selective pressure that aids drug resistance, and the likelihood of occurrence is increased by prolonged use of the antibiotics. The likelihood of developing resistance increases when antimicrobial drugs are used for a longer duration [67,71]. Bacteriostatic drugs, which do not kill bacteria but inhibits them, gives an opportunity for the regrowth of some bacterial cells and thus they develop resistance when exposed to the drug. An insufficient number of doses or missed scheduled doses (as a result of poor patient compliance) gives ample time for the development/acquisition of resistance genes [72].

**Figure 4:**
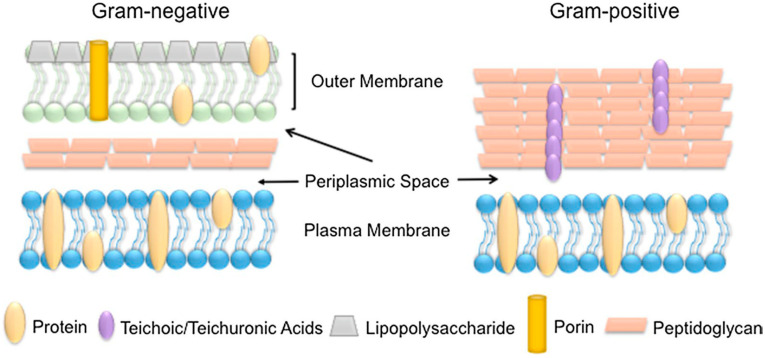
Comparison of the bacterial cell wall structure (adapted with permission from [20], BioMed Central, 2017).

Bacteria utilize several mechanisms for resisting antimicrobials. Of such, the mechanism is the decreased uptake and increased efflux of the drug from the bacterial cell. This happens by the transmembrane efflux pump that prevents the antimicrobial agent from attaining the toxicity level within the bacterial cell [7,71]. The low sensitivity of *P. aeruginosa* and *E*. *coli* to antibiotics is due to their drug efflux system. Both are Gram negative bacteria having a distinct outer membrane enclosing a periplasmic space. This periplasmic space contains a peptidoglycan cell wall that envelopes an inner membrane (Figure 4). It was reported that the drug efflux pump of *P*. *aeruginosa* contains an inner membrane H+/drug antiporter protein bound to a linker protein in the periplasmic space, which itself is bound to an outer membrane channel protein [73]. An over expression of these efflux proteins was seen in *P. aeruginosa*. This is usually seen when there is a mutation of the regulatory protein that is required to suppress genes coding for efflux proteins [73]. *E*. *coli* also uses the mechanism of drug efflux. It expresses a minimum of at least nine pumps whose energy source to expel different types of antibiotics is the transmembrane proton gradient. Thus, conferring multidrug resistance to *E. coli.* This drug efflux system is commonly seen in Gram −ve bacteria because their additional outer membrane consists of a lipopolysaccharide compared with Gram +ve bacteria with a peptidoglycan cell wall surrounding only a single plasma membrane (Figure 4). This explains why Gram −ve bacteria are less susceptible to many antibiotics compared with Gram +ve bacteria [20,67,73]. Other mechanisms of antibiotic resistance are by enzymatic inactivation of the antibiotic, covalent modification of the drug, mutation of antibiotic targets, protection of targets, etc., as seen in the case of methicillin resistant *Staphylococcus aureus* (MRSA) and *Klebsiella pneumoniae* [67,69,73].

Few studies have reported the resistance of bacteria to MNPs and has been attributed to the development of extracellular substances that leads to agglomeration and precipitation of the MNPs [74,75,76,77]. Recently, the exposure of *E. coli* 013, *E. coli* CCM 3954 and *P. aeruginosa* to subinhibitory concentrations of AgNPs was studied. Several interactions with the AgNPs led to the development of antibiotic resistance. The continuous exposure provided time for the bacteria to develop a counter mechanism against the effect of the AgNPs. This was achieved by the secretion of flagellin, an adhesive protein of the bacteria flagellum, which reduces the stability of the AgNPs and causes its aggregation and precipitation. Thus, preventing the entry of the AgNPs into the bacteria cell and therefore loss of antibacterial activity [78]. Formation of the biomolecule corona is another mechanism through which bacteria develop resistance to MNPs. This often happens in the physiological environment such as the gastrointestinal tract, lungs and wounds. The produced biomolecule corona hinders the binding of the nanoantibiotic to the pathogenic bacteria [79]. In addition, resistance to bactericidal effects of MNPs has been reported to occur due to the ability of the bacteria to alter its surface charge as a defense mechanism. This is done by altering the phospholipid structure, which changes the electrical charge on the surface of the bacteria [80,81]. However, recent studies of the inhibitory role of the pomegranate rind extract against the production of flagellin offers the significance of plant mediated MNPs as a means of combatting antibiotic resistance [78].

## 4. Overview of the Bactericidal Mechanism

There are diverse proposed mechanisms through which MNPs exert a bactericidal effect and combat antimicrobial resistance against Gram −ve and Gram +ve bacteria. Noble MNPs such as Au, Ag and its bimetallic are known to act as potent broad-spectrum antimicrobial agents. The bactericidal effect of these MNPs usually results from mechanisms such as release of metal ions, cell wall and membrane damage, intracellular penetration and DNA damage, generation of reactive oxygen species (ROS), lipid peroxidation, ATP depletion and damage of biomolecules.

### 4.1. Cell Membrane: Lipid and Protein Interaction

**Figure 5:**
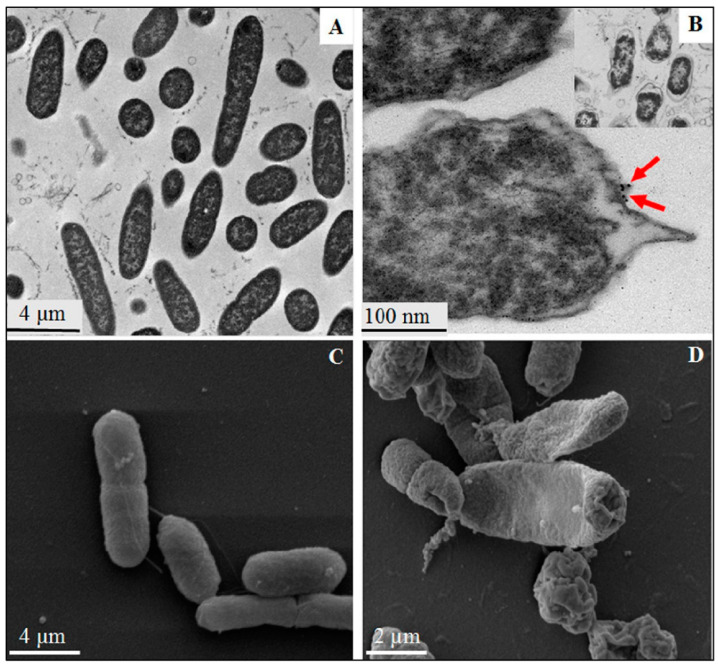
TEM (**A**,**B**) and SEM (**C**,**D**) images of *E. coli* O157:H7 cells: (**A**,**C**): untreated cells (control groups) and (**B**,**D**) cells treated with different concentrations of silver nanoparticles (AgNPs) adapted with permission from [94], Elsevier, 2018.

**Figure 6:**
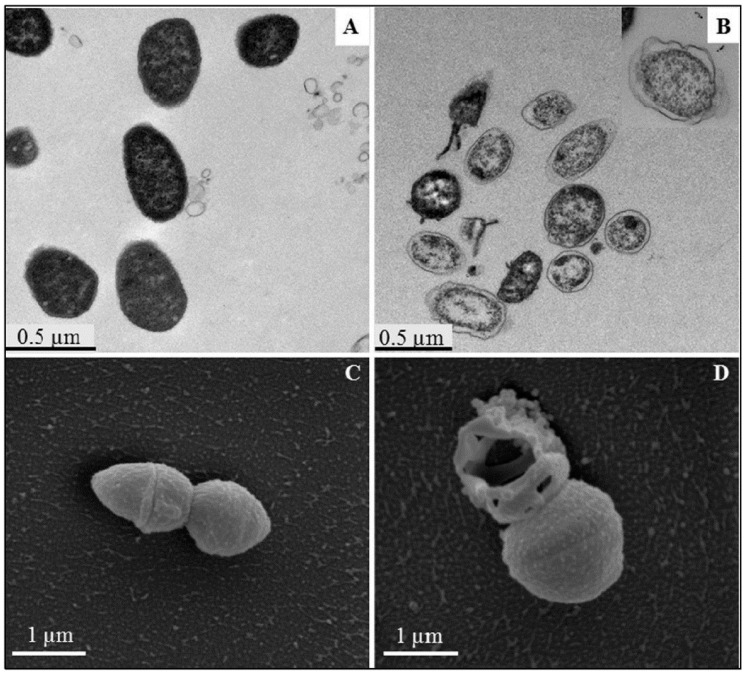
TEM (**A**,**B**) and SEM (**C**,**D**) images of *L. monocytogenes* cells: (**A**,**C**) untreated cells (control groups) and (**B**,**D**) cells treated with different concentrations of AgNPs, adapted with permissions from [94], Elsevier, 2018.

Metal NPs gradually discharge metal ions that is able to cross the membranes and disrupt cellular processes from inside the cell [82]. Different adhesion pathways are available for the attachment of NPs to the cell wall and membrane. These barriers serve to protect the microorganism against external threats and to maintain homeostasis while still permitting the transport of nutrients inside the cell. The classification of bacterial is based on the differences in the structure of their cell wall. The cell wall (envelope) of Gram −ve bacteria has a minimum of two layers of lipopolysaccharides. On the other hand, that of Gram +ve bacteria is basically thicker. Gram +ve bacteria has a thick layer of peptidoglycan within their cell walls while Gram −ve bacteria have a thin layer of peptidoglycan with an extra outer membrane embedded lipopolysaccharide. This additional membrane in Gram −ve bacteria means that there is also an extra membrane layer termed periplasm (Figure 4). Several research works have reported that Gram +ve bacteria are more resistant to MNPs mechanisms of action [83,84,85,86,87]. This is due to the different cell wall structure. In Gram −ve bacteria, such as *E. coli*, a 1–3 μm layer thick of lipopolysaccharides cover the cells, in addition to 8 nm thick layer of peptidoglycans. This facilitates the passage of ions from NPs into the cell whereas Gram +ve bacteria like *S*. *aureus* have a thicker peptidoglycan layer, which stretches over 80 nm with covalently bound teichoic and teichuronic acids. The damage to the cell membrane of bacteria that happens from the interaction between the cells and MNPs becomes more harmful to the Gram-negative bacteria. This is due to the absence of a thick protective layer of peptidoglycan as seen with Gram +ve bacteria Furthermore, Gram −ve bacteria susceptibility to MNPs is due to their negatively charged lipopolysaccharide. This causes an attraction to the positive ions released by most MNPs. The consequent effect is an accumulation of ions that leads to intracellular damage. However, it is known that both Gram +ve and Gram −ve bacteria have a negatively charged cell wall that allows for interactions between the cell wall and the MNPs or its ions [88]. A study of Gram −ve *Salmonella typhimurium* revealed that a mosaic of anionic surface domains present on the cell wall in an abundant measure [89]. Thus, increased toxicity is observed when a high concentration of NPs binds to these negative anionic domains. Additionally, through mathematical calculations and electrophoretic mobility study, it was found that *E. coli* is more negatively charged and rigid than *S. aureus* [90]. The outer membrane comprising of proteins and lipids is the first barrier encountered by AgNPs. Silver forms a complex with electron donors like nitrogen, oxygen, sulphur atoms or phosphorus in the interactions with the proteins in the outer membrane. This interactions leads to the inactivation of proteins and membrane bound enzymes of the bacterial cell wall [91,92,93]. The bactericidal mechanism of AgNPs biosynthesized with turmeric against *E. coli* O157:H7 and *Listeria monocytogenes* was elucidated by Alsammarraie et al. [94]. Microscopic images of cells treated with AgNPs showed cell membrane damage with irregular shapes, protrusions and fragmentations. The cytoplasmic membrane of both cells was separated from their cell walls and completely damaged. This led to their rupture and release of cell constituents due to the physical impacts of the AgNPs. In addition, deposits of AgNPs were seen around severely damaged bacterial cells, both in the cell membrane and cytoplasm of the bacteria, especially in *E. coli* O157:H7 (Figure 5B). The treated cell shown in Figure 5D and Figure 6D reveals a big hole and fragmented cell membrane that resulted in a totally lysed cell. Figure 5D further reveals severe shrinkage cytoplasmic constituents’ leakage of *E. coli* O157:H7. The TEM and SEM micrographs confirm that the antibacterial activity of biosynthesized AgNPs by turmeric was obviously bactericidal and not bacteriostatic. SEM-EDS analysis showed that a strong signal of elemental Ag was present in the treated cells confirming that AgNPs were responsible for the observed damages in the cells. The Fourier transform infrared (FTIR) microspectroscopic method was used to study the bactericidal mechanism of garlic acid (Ga) conjugated AuNPs (AuNPs-Ga) against *Plesiomonas shigelloides* and *Shigella flexneri* [95]. The results were analyzed by a principal component analysis (PCA). There were two regions of interest in the PCA. Firstly was the biochemical print region for stretching vibrations of esters found in lipids (1800–1000 cm^−1^), amide I and II groups belonging to peptides and proteins (1655–1637 cm^−1^), P=O stretching of nucleic acids (1250–1220 cm^−1^ and 1084–1088 cm^−1^) and the typical bands for polysaccharides and carbohydrates (1200–900 cm^−1^). Secondly was the wavenumber from 3000 to 2850 cm^−1^ that is attributed to known functional groups of specific amino acid side chains and membrane fatty acids. The PCA plot revealed the differences in the spectra of the treated and untreated bacterial cells. Each of the representative loading plots had a change in their lipid, protein and cellular phosphorylation signal [95,96,97,98]. Significant changes in the lipid and protein signal signifies a destruction of the cell membrane and biochemical alteration of the bacteria cells.

### 4.2. Free Radical Generation

Biological molecules such as lipids, proteins and nucleic acids are adversely affected by free radicals. This causes alteration of the normal redox status and lead to increased oxidative stress [99]. Although, oxidative stress is a normal cellular process that occurs in several phases of cellular signaling however, extreme oxidative stress can be detrimental. Literature has showed that MNPs can trigger cellular oxidative stress [100,101,102]. These free radicals are either in the form of reactive oxygen species (ROS) or reactive nitrogen species (RNS). They result from either endogenic sources (endoplasmic reticulum mitochondria, peroxisomes, etc.) or exogenic sources (heavy metals, pollution, transition metals and specific drugs) [99].

When cells are exposed to stress, they show defensive responses via enzymatic or non-enzymatic mechanisms [102,103]. Damage to the DNA, cell wall, proteins and lipids usually occurs when the defense mechanism is overpowered by oxidative stress. Free radicals such as singlet oxygen, hydrogen peroxide (H_2_O_2_) and hydroxyl radical (-OH) are released when the defense mechanism is weakened by the oxidative stress. All these can lead to lipid oxidation, which inhibits or kills bacteria growth. Cell membranes can easily be disrupted by both endogenic and exogenic ROS [104,105]. Chakraborty et al. [106] evaluated the antimicrobial effects of *Thevetia peruviana* mediated AgNPs on *E. coli.* The as-synthesized AgNPs showed an effective inhibitory effect against *E. coli* with an inhibition zone of 20 mm. This suggests that the antibacterial potency of the AgNPs might be related to the membrane structure of the bacteria. Electron spin spectroscopy (ESR) was used to investigate if the free radical production from AgNPs formed at pH 7 after 48 h of reaction time is related to the antimicrobial activity. The results showed that the growth inhibition was due to the formation of free radical species from the surface of AgNPs, which altered the permeability of the outer membrane and inactivated the respiratory function of the bacteria.

Soo-Hwan et al. [107] showed that the mechanism of bactericidal effect of AgNPs against *S. aureus* and *E. coli* was by the production of ROS due to increased membrane permeability and the inactivation of lactate dehydrogenase, which eventually led to protein breaks. More protein leakage occurred in the membrane of *E. coli* compared with that *S. aureus*. This observed difference was possibly attributed to the thickness of the peptidoglycan layer of *S. aureus* [107]. Gomaa [108] corroborated these results in the study of the bactericidal mechanism of AgNPs with respect to *S. aureus* and *E. coli*. The growth curve was measured, followed by an estimation of the protein and reducing sugar leakage. Furthermore, lethal ROS and respiratory chain dehydrogenase activity were evaluated. The study showed that 50 mg/mL AgNPs completely inhibited the growth of bacterial cells and damaged the bacterial membrane permeability, depressing the activity of some membranous enzymes, which eventually led to bacteria cell death. In this study, Dye 20, 70-dichlorofluorescein diacetate (DCFH-DA) was used to measure the ROS. It was observed that after 6 h incubation of the *E. coli* and *S. aureus* with AgNPs, there was a significant increase in ROS production however, this was not observed in the control groups. Significantly, AgNPs are stress inducers for bacteria.

**Figure 7:**
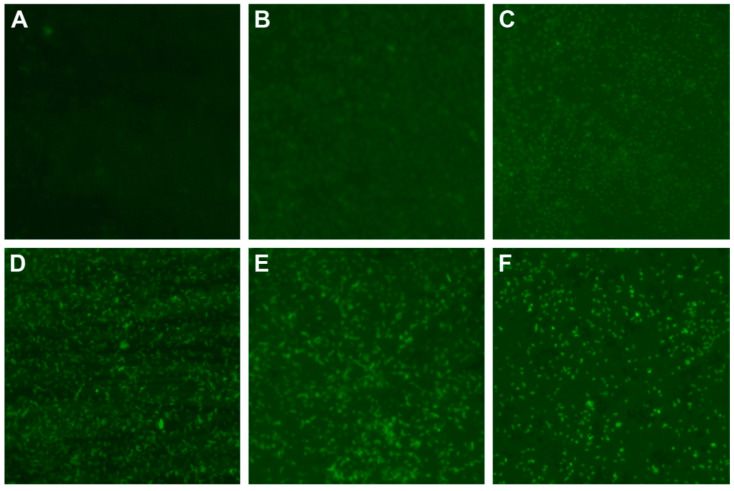
Changes of reactive oxygen species (ROS) production in AgNPs-treated multidrug-resistant *Pseudomonas aeruginosa* at different time intervals under fluorescence microscopy with ×400 magnification. Notes: (**A**) the untreated *P. aeruginosa* without observable fluorescence. (**B**–**F**) Fluorescence observation of the bacteria treated with AgNPs at different points of 0.5, 0.75, 1, 1.5 and 2 h, respectively, indicating that AgNPs induce ROS production in a time-dependent manner) adapted with permission from [112], Dove Medical Press, 2019. Abbreviations: AgNPs—silver nanoparticle; ROS—reactive oxygen species.

**Figure 8:**
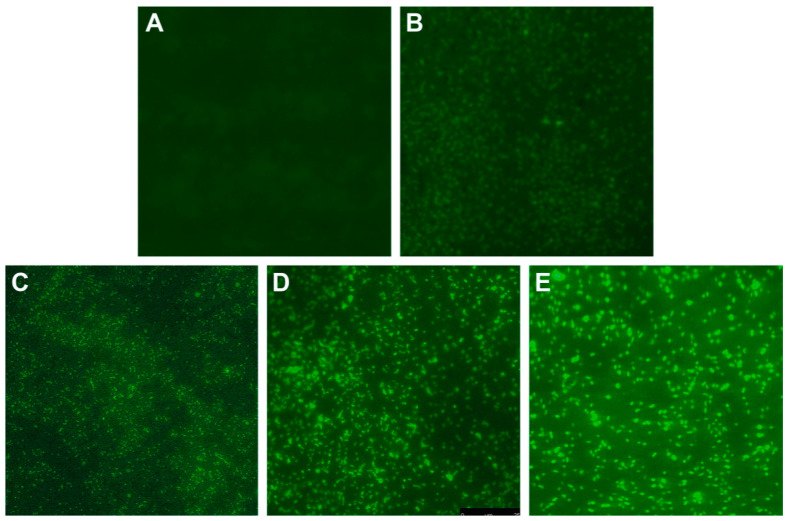
Changes of ROS production in multidrug-resistant *Pseudomonas aeruginosa* exposed to different concentrations of AgNPs under fluorescence microscopy with ×400 magnification. Notes: (**A**) the untreated *P. aeruginosa* without observable fluorescence. (**B**–**E**) Fluorescence observation of the bacteria exposed to 5.625, 11.25, 22.5 and 45 μg/mL AgNPs, respectively, adapted with permission from [112], Dove Medical Press, 2019. Abbreviations: AgNP, silver nanoparticle; ROS, reactive oxygen specie.

Qayyum et al. [109] expanded their study to Gram negative (*K. pneumoniae*, *P. aeruginosa* and *E. coli*) and Gram positive (*S. mutans* and *S. aureus*) strains. The results showed that green AgNPs produced ROS after 4 h of incubation with the bacterial cells. It was observed that increased contact time of the AgNPs with the bacterial cells led to increased production of the ROS. Additionally, the quantity of ROS increased several times compared to that of the control group for both Gram positive and Gram negative bacteria. However, more ROS production was observed in the treated Gram negative *E. coli* bacterial cells compared to treated Gram positive *S. mutans* bacterial cells [110]. ROS formed as a result of bacterial interactions with AgNPs that causes damage to the bacterial cell membrane, protein structure and intracellular systems. In studying the conditions and mechanism of antibacterial activity of silver nanoparticles (AgNPs) against *E. coli* O157:H7 (CMCC44828), it was also established that the presence of oxygen generated more ROS, which led to increased antimicrobial activity [111]. The mechanism of antibacterial activity of AgNPs against multidrug resistant *P. aeruginosa* was studied by using H2DCF-DA staining and fluorescence microscopy [112]. It was observed that there was an increasing intensity in the fluorescence of the bacteria treated with AgNPs with increased time while the untreated bacteria had a weak fluorescence (Figure 7). In a similar manner, the fluorescence of the treated bacteria increased with increased concentration of AgNPs in a 1 h exposure period (Figure 8). Thus, it was deduced that AgNPs induced an excess generation of ROS in multidrug resistant (MDR). This was based on a time and concentration dependent manner.

### 4.3. DNA Damage

**Figure 9:**
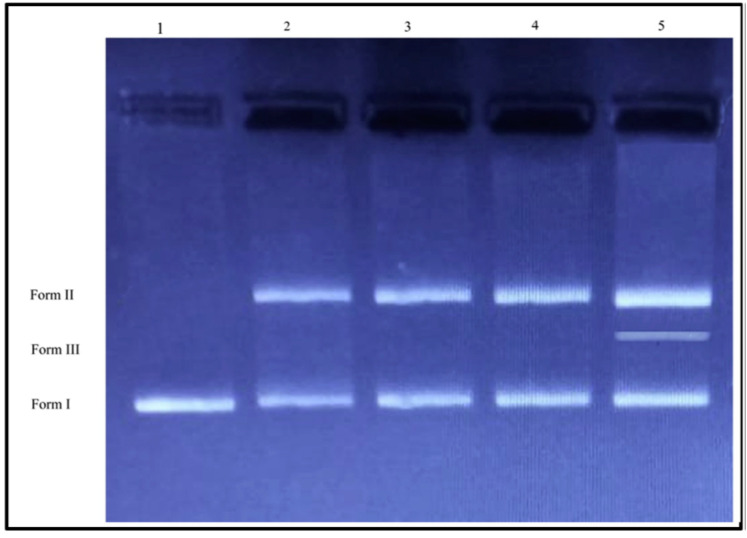
DNA cleavage activity of Rc-AgNPs. Lane 1, pBR 322 DNA; Lane 2, pBR 322 DNA + 100 mg/L of Rc-AgNPs (60 min incubation); Lane 3, pBR 322 DNA + 200 mg/L of Rc-AgNPs (90 min incubation); Lane 4, pBR 322 DNA + 100 mg/L of Rc-AgNPs (60 min incubation) and Lane 5, pBR 322 DNA + 200 mg/L of Rc-AgNPs (90 min incubation) Rc = *Rosa canin*, adapted with permission from [116], Elsevier, 2019.

The DNA of any organism stores genetic information about the cell. Damage to the DNA can either result in mutation or cell death. Cui et al. [113] studied the mechanism of the bactericidal effect of AuNPs on *E. coli* (ATCC 11775) through transcriptomic and proteomic approaches. The antibacterial mechanism of AuNPs was found to be due to (i) a change in membrane potential and inhibition of ATPase activities that led to a decrease in ATP level thus a reduction in metabolism and (ii) inhibition of the subunit of ribosome for tRNA binding. Their finding showed that the bactericidal action was not due to ROS. This might be novel to developing bactericidal MNPs, which aims at the energy metabolism and transcription of bacteria without generation of ROS species that could be harmful to the mammalian host of the bacteria. The antibacterial mechanism of AgNPs on the clinical isolates of *P. aeruginosa* and *S. aureus* was studied by Abbas et al. [114]. This was done by analyzing the effect of AgNPs on the bacterial genome by estimating the amplification of AgNPs treated or untreated bacteria with DNA by real-time PCR. The results by real-time PCR showed damage in the DNA of *P. aeruginosa*. This was exemplified by the delay in the amplification of the exoA gene in the treated sample compared to the control sample. In addition, this also lowered amplification efficiency in AgNPs bacteria as compared with the untreated bacteria. This clearly shows that the mechanism of the bactericidal effect of biosynthesized AgNPs on *E. coli* and *S. aureus* can been attributed to DNA cleavage activity [115]. DNA as the genetic information unit of all livings organisms is fundamental to functional existence. An alteration in the genetic composition opens the pathway to malfunctioning and eventually cell death. In recent times the DNA cleavage ability of *Rosa canin*-AgNPs was investigated using agarose gel electrophoresis [116]. A difference in band was observed in the treated plasmids with AgNPs compared to that of the control DNA (Figure 9). It was noticed that plasmid pBR322 changed from Form I into Form II for Lanes 2–4. Furthermore, at a concentration of 200 mg/L for 90 min, the AgNPs served as chemical nucleases by cleaving the DNA Form I into Form III (lane 5). Conclusively it can be said that the AgNPs exerted a bactericidal effect by cleaving the genome of the pathogenic microorganism.

## 5. Conclusions and Future Perspectives

Green synthesis of nanoparticles can serve as a future direction in biomedical nanotechnology for the development of effective antimicrobial compounds. It has been established in the literature that MNPs exhibit strong antibacterial activity. Multiple pathways simultaneously activated by NPs make their exposure to bacteria cells effective and this is promising to combat antibiotic resistance. The production of ROS, cell wall penetration, DNA damage and metabolite binding are mechanisms evasive to bacteria’s defense systems. Most research in the biosynthesis of MNPs uses the whole plant extracts as a bioreductant and stabilizer. However, identification of the pure biomolecule or compound responsible will help optimize the synthesis and its antibacterial application. This will provide an opportunity to understand the bactericidal mechanism of MNPs at the molecular level. To address the emerging number of multiple-drug resistant bacterial strains, more clinical strains should be tested rather than evaluation of traditional strains from microbial collections. Relentless efforts from researchers in advancing NPs synthesis and its applications have offered the possibility to future alternatives in biomedical applications, pharmaceutical, theragnostic and therapeutics. Aside from this, the studies showed that MNPs have a potential to be the choice solution in antibacterial applications in the near future.
